# A Review on the Use of Metal Oxide-Based Nanocomposites for the Remediation of Organics-Contaminated Water via Photocatalysis: Fundamentals, Bibliometric Study and Recent Advances

**DOI:** 10.3390/toxics11080658

**Published:** 2023-08-01

**Authors:** Evando S. Araújo, Michel F. G. Pereira, Georgenes M. G. da Silva, Ginetton F. Tavares, Carlos Y. B. Oliveira, Pedro M. Faia

**Affiliations:** 1 Research Group on Electrospinning and Nanotechnology Applications, Department of Materials Science, Federal University of San Francisco Valley, Juazeiro 48902-300, Brazil; michel.pereira@discente.univasf.edu.br; 2 Federal Institute of Education, Science and Technology of the Sertão Pernambucano, Petrolina 56314-520, Brazil; georgenes.gil@ifsertao-pe.edu.br; 3 Research and Extension Center, Laboratory of Fuels and Materials (NPE/LACOM), Department of Chemistry, Federal University of Paraíba, Campus I, João Pessoa 58051-900, Brazil; ginetton@reitoria.ufpb.br; 4 Laboratory of Phycology, Department of Botany, Federal University of Santa Catarina, Florianópolis 88040-535, Brazil; yure.oliveira@ufsc.br; 5 Electrical and Computer Engineering Department, Centre for Mechanical Engineering, Materials and Processes (CEMMPRE), FCTUC, University of Coimbra, Polo 2, Pinhal de Marrocos, 3030-290 Coimbra, Portugal; faia@deec.uc.pt

**Keywords:** metal oxide, nanocomposites, organic pollutants, water remediation, heterogeneous photocatalysis

## Abstract

The improper disposal of toxic and carcinogenic organic substances resulting from the manufacture of dyes, drugs and pesticides can contaminate aquatic environments and potable water resources and cause serious damage to animal and human health and to the ecosystem. In this sense, heterogeneous photocatalysis stand out as one effective and cost-effective water depollution technique. The use of metal oxide nanocomposites (MON), from the mixture of two or more oxides or between these oxides and other functional semiconductor materials, have gained increasing attention from researchers and industrial developers as a potential alternative to produce efficient and environmentally friendly photocatalysts for the remediation of water contamination by organic compounds. Thus, this work presents an updated review of the main advances in the use of metal oxide nanocomposites-based photocatalysts for decontamination of water polluted by these substances. A bibliometric analysis allowed to show the evolution of the importance of this research topic in the literature over the last decade. The results of the study also showed that hierarchical and heterogeneous nanostructures of metal oxides, as well as conducting polymers and carbon materials, currently stand out as the main materials for the synthesis of MON, with better photocatalysis performance in the degradation of dyes, pharmaceuticals and pesticides.

## 1. Introduction

The increase in industrial and agricultural production, combined with the accelerated growth of the world population in recent decades, are some of the main factors that contribute to the pollution of the physical and biological components on Earth [1,2].

For instance, plastic, textile, pharmaceutical and pesticide products consumption has grown exponentially, being vital for society, but is seriously contributing to fast-growing pollution worldwide [1,2,3]. In other words, wastewater with organic pollutants must primarily undergo decontamination treatments and meet the purity standards required by government agencies before being discharged into waterways [3,4,5].

Primary treatment of wastewater, using filtration and sedimentation, generally captures only larger particles. The smaller particles remain in the water, with the need for additional costs in a water treatment plant to arrange other decontamination methods in order to lessen the problem. Among the alternative techniques, it is possible to mention chemical oxidation, other filtration processes (using sand and biological filters), membrane separation, adsorption of the compounds with activated carbon, and ozonation [5].

The scarcity of effective and low-cost procedures that ensure large scale discard and treatment of manufacturing wastes (due to their toxicity and carcinogenic composition resulting from the decomposition of its molecules) is a limitation for the decontamination of aquatic environments and potable water, vital for life preservation [1,4,5]. All these substances negatively interfere in the life cycle of species, and in the availability and quality of vital elements for living beings [4,6]. For example, the contamination of aquatic systems (rivers, lakes, effluents, etc.) by organic compounds can result in imminent risks to human health. Ingestion and/or skin contact with organics-contaminated water can involve diseases, such as diarrhea, motion sickness, dermatitis, malnutrition and even cancer [4]. In this sense, the search for new technologies and functional materials for the treatment of organics-contaminated waters, which are able to meet current needs, with lower cost and environmental impact, is an open field of research today [6,7,8].

Metal oxide nanostructures (materials that respond to external stimuli like light, electric field, magnetic field, pressure, to mention only a few) have gained increasing attention from academic researchers and industrial developers, as potential materials to produce efficient and environmentally friendly devices for the remediation of water contamination by organic compounds [9,10,11,12]. Currently, among the techniques for removing contaminants using metal oxides, heterogeneous photocatalysis stands out. These applications are directly related to metal oxide nanostructures properties, such as: high chemical and structural stability; high surface area/volume ratio; excellent electrical/electronic bulk and electric charge transfer properties (electrons (e^−^) and holes (h^+^)); easy surface interactivity with contaminant molecules; and the possibility of reusing the material in new decontamination cycles [13].

In addition, metal oxides can form nanocomposites with other oxides and other functional materials, such as conducting polymers and carbon-based materials, in order to ensure greater surface area/volume ratio, lower electron-hole recombination and better adsorption, photocatalysis and sensing performances, when compared to results reported for pure oxides [13,14,15]. These nanostructured materials allow for a wider range of applications considered an active research object today.

This work presents an updated review on the use of metal oxide-based nanocomposites photocatalysts for remediation of organics-contaminated water. A bibliometric analysis is also presented, showing the evolution of this research topic in the literature over the past decade. In addition, recent scientific contributions to the area are presented and discussed based on the performed analysis.

## 2. Metal Oxides and Photocatalysis

### 2.1. Fundamentals of Metal Oxides

Metal oxides belong to the group of semiconductor materials, which have chemical structures formed by the interaction of charges belonging to oxygen atoms (highly electronegative) with atoms of metallic elements. By definition, semiconductors have a structure composed of electronic bands, where the valence band (filled) is separated from the conduction band (unfilled) by a bandgap, with sufficient energy (around 1.0–3.0 eV) to maintain them with electrical insulators behavior at the temperature of 0 Kelvin [16,17].

Metal oxides gained attention in the 1950s due not only to their potential to catalyze acid-base reactions, but to their potential participation with double acid-base sites in catalytic events (small regions that have the simultaneous participation of an acid and a base) [18]. In general, the catalytic surface of metal oxides consists of anionic oxygen centers, as Lewis base sites (O^2−^), and of coordinatively unsaturated cationic metal centers, as Lewis acid sites. Anionic oxygen centers have high electronegativity and can form ionic bonds when interacting with metals.

These materials can be produced through various industrial methods, such as chemical treatments of their precursor reagents, hydrothermal and precipitation reactions, sol-gel, sputter-coating, green synthesis, among others [19].

With these synthesis routes, it is possible to produce metal oxides with particle sizes on the nanometric scale (of the order of 10^−9^ m), which directly influences the increase in the surface area/volume ratio of these materials and, consequently, the greater efficiency in the application of these oxides as functional materials.

These materials present characteristics of conductors when they are excited by external environmental stimulus (such as light, pressure, electrical field, heat, magnetic field, etc.) with enough energy to allow electrons (e^−^) of the valence band to migrate to the conduction band, jumping over the bandgap (consequently generating active holes (h^+^) in the valence band) [16,17]. In other words, their electrical and electronic properties are directly dependent on their electronic bands’ energy structure.

In this context, metal oxide nanostructures stand out in relation to other materials due to their synergistic combination between high superficial area/volume ratio and tunable optical, magnetic, electrical, mechanical, thermal, catalytic and photochemical characteristics, all resulting from their bulk and surface [20]. These properties combined with their excellent chemical, structural and environmental stability, ensure them a variety of emerging applications, such as the production of electrochemical, optical and gas sensors, fuel cells, solar cells, piezoelectric devices, supercapacitors, adsorbents, photocatalysts, just to mention a few [20,21,22].

The surface morphology of most metal oxides is critical in a variety of chemical processes. Metal oxides have been widely studied due to their optical, electronic, magnetic, electrical and mechanical characteristics, in addition to their high chemical stability and resistance to high temperatures (higher than the observed for polymeric materials), making them suitable for various applications in Science & Technology [21].

Gas sensing [23] and environmental remediation [24] by means of photodegradation of organic dyes and heavy metals in aquatic media are examples of processes that can be performed and/or improved by enhancing metal oxides surface interaction with the media [25].

When these materials are available in nanometric scale, they may present improved physical and chemical properties, such as adsorption capabilities, diffusion, surface area and modifiable surface functionalities, physicochemical stability, among others [20,22].

Metal oxide-based nanocomposites (MON) are materials that are originated in the interaction between two or more metal oxides, or between metal oxides and other functional nanomaterials. Conducting polymers, metal nanoparticles and carbon-based materials are some examples of functional materials that are known to improve metal oxides properties [22,26,27]. The main objective of this interaction is to provide enhanced attributes to the resulting material in comparison to those presented by single oxides, such as greater chemical/structural stability, lower bandgap, electron/hole recombination reduction, and greater contact area [13].

The possibility of producing metal oxide-based nanocomposites with controlled size, morphology, composition, and interaction with other structures, will allow one to improve the just mentioned characteristics, opening simultaneously new perspectives for environmental applications, such as more efficient photocatalysis processes used for decontamination of aqueous media.

### 2.2. Electronic Structure

**Figure 1:**
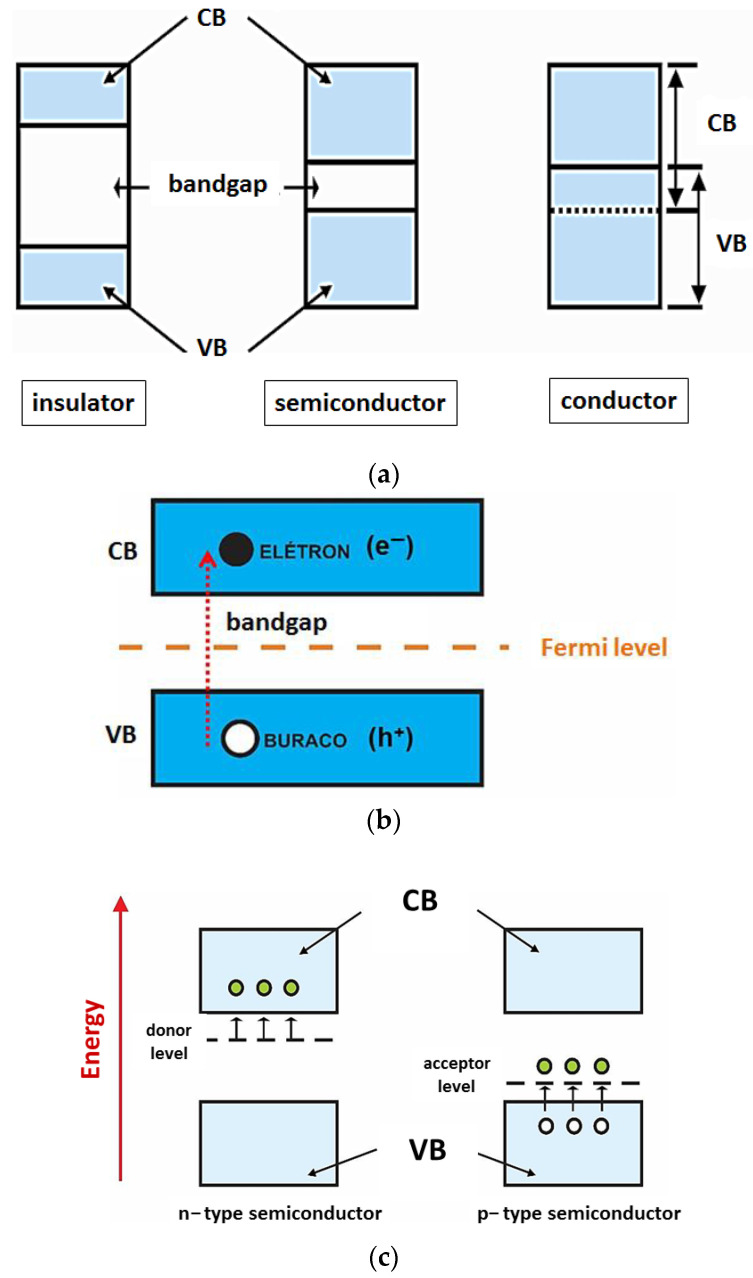
(**a**) Energy band model for insulating, semiconducting and conducting materials. (**b**) Illustration of the excitation of an electron from VB to CB, highlighting the generated charge carriers (e^−^ and h^+^). (**c**) Energy bands of n−type and p−type extrinsic semiconductors.

Metal oxides exhibit changes in their electrical/electronic properties based on variations in their crystalline structure, promoted by doping processes and/or external stimuli, such as electrical field, temperature, light, etc. These properties are based on the Band Theory (proposed by the physicist Felix Bloch, in 1928), which states that in a system with N identical atoms, each of the atoms orbitals gives rise to N new orbitals, thus forming a set of N levels with very close energy. The formed set is known as energy bands (Figure 1a) [28].

According to this model, metal oxides as well as all other semiconducting materials possess two well-defined energy bands: the valence band (VB) and the conduction band (CB) (Figure 1a). The band formed by the energy levels occupied by electrons, in the lower layers of the electronic distribution, is named the valence band, while the energy levels non-occupied by electrons in the higher layers form the conduction band. Between the VB and the CB, there is a quantum forbidden energy region (where no energy levels can be filled by electrons), called bandgap or forbidden band, which width is known as Eg. The energy dimension of this region determines whether a material is a conductor, semiconductor or insulator [28,29].

In other words, the bandgap determines the minimum energy needed to excite the electrons from the VB to the CB, with a wavelength (λ) in the range of the ultraviolet–visible radiation spectrum [30]. For insulating materials, the bandgap energy is greater than 5 eV and the density of free charge carriers in the CB is extremely low. In the case of semiconducting materials, the bandgap is much narrower (less than 5 eV): thus, they can change their condition from insulator to conductor by absorbing energy from irradiated photons, which possess energy greater than that of the bandgap. Consequently, VB electrons acquire enough energy that allows them to reach the CB (in the condition that they are free electrons, e^−^), which provides the semiconductor characteristics of a conductive material [28,29]. The excitation of an electron from the VB to the CB generates a hole (or hole, h^+^) in the VB, which behaves like a positively charged particle. As a consequence, two charge carriers are generated, an electron (e^−^) and a hole (h^+^) (Figure 1b): this phenomenon is known as photoexcitation [31].

In these materials, photoexcitation can be hindered by electron/hole recombination. When recombination occurs, the hole is undone. On the other hand, the continuous incidence of an external stimulus on the material (a light source, for example), with energy greater than the bandgap of the material, can produce new holes in the VB, raising the electrons from the VB to the CB. Thus, it is possible to reduce the rate of the electron/hole recombination, therefore improving photocatalysis processes, for example, which directly depend on the interaction of the electric charge carriers with the contaminant molecule atoms.

Semiconductors can also be classified as intrinsic or extrinsic, due to the absence or presence of impurities in their crystalline lattice, respectively [32]. Intrinsic semiconductors have the same amount of positive and negative carriers, since, for each electron that is promoted to the valence band, a hole is generated in the conduction band.

Thus, the electrical conductivity of the intrinsic semiconductor is defined by the product of the number of electrons and holes, by their respective charges and mobilities. Positive charges move in the same direction as the one of the electric field, while negative ones move in the opposite direction.

Another important concept for understanding the mobility of charge carriers in these materials is the Fermi energy level [33], located in the bandgap region (Figure 1b) of the semiconductors. The Fermi level represents the highest energy level, at absolute zero temperature, that an electron can occupy [33]. For an intrinsic semiconductor, the Fermi level is exactly in the center of the bandgap. Extrinsic semiconductors are characterized by defects in the crystalline lattice (such as the absence of some atoms or their positioning outside the original position) and/or the presence of other elements through donor or acceptors doping (insertion of small amounts of substitutional atoms) [34].

These defects allow energy levels in the bandgap to act as traps for charge carriers that are free in the crystalline structure, shifting the Fermi level position within the bandgap. Thus, the doping of a semiconductor with host atoms modifies the electronic distribution of electrons. For an extrinsic n-type semiconductor, the Fermi level shifts to a position just below the conduction band, whereas for p-type semiconductors, this level appears just above the top of the valence band [35] (Figure 1c, white balls represent holes and green balls electrons).

In the case of donor dopants (n-type semiconductors), atoms with a higher number of valence electrons are added (insertion of a group V atom into a crystal dominated by group IV atoms, for example). In this configuration, the semiconductor acquires an excess of electrons in relation to the number of holes. In this context, room temperature energy is sufficient for detaching an electron from the donor intermediate level (located in the bandgap), promoting it to the conduction band. In other words, the binding energy of the donor impurity is lower, which causes the donor energy level to be slightly below the conduction band of the host material. Analogously, in the case of acceptors (p-type dopants), atoms with less valence electrons are inserted (for instance, insertion of a group III element atom into a crystal composed essentially of group IV atoms). The absence of an electron Is treated as a hole In the valence band [36].

This process alters the electronic density of the valence (p-type semiconductor) or conduction (n-type) bands: consequently, the new possibly occupied energy levels cause the bandgap energy to shrink, with a consequent increase in the electrical conductivity of the material. Siu’s work [36] explains that the type and quantity of added impurities depends on the form and conditions of their growth, whether synthetically or naturally. In resume, by introducing impurities into the metal oxide crystalline network, it is possible to obtain an increase in the number of charge carriers and to improve the electronic properties of the final material for photocatalysis applications.

In the literature, several semiconducting metal oxides, such as titanium dioxide (TiO_2_), zinc oxide (ZnO), tin dioxide (SnO_2_), tungsten trioxide (WO_3_), niobium pentoxide (Nb_2_O_5_), indium oxide (In_2_O_3_), cerium oxide (CeO_2_) and graphene oxide have been explored, at the nanoscale level, for the development of functional photocatalysis devices [13].

The state of the art on the subject confirms that the electronic and microstructural properties of metallic oxides-based photocatalysts devices were improved when used at their nanocomposite level: their synthesis at this level resulted from the interaction between two or more metal oxides or between metal oxides and other functional components/materials such as metal nanoparticles, conductive polymers or carbon materials. Sensors, fuel cells, supercapacitors, solar cells and photocatalysts are some of the examples of more recent technological applications for which significant enhancements were observed and reported [37,38].

### 2.3. Metal Oxide-Based Photocatalysis

The term photocatalysis means to increase the kinetics of a photoreaction by the action of a catalyst. Photocatalysis is a photochemical process, of the advanced oxidative processes group, for the formation of free radicals, which are highly oxidizing and degrade organic substances in water.

Photocatalysis processes can be homogeneous or heterogeneous. The heterogeneous photocatalysis process differs from the homogeneous one, due to the former’s particularity of using catalysts in the solid state and, consequently, radicals are generated from the reactions between electron/hole (e^−^/h^+^) pairs with the medium, under the action of an incident light. This radiation is necessary for the homolytic breakdown of the oxidant to occur [39]. The first heterogeneous photocatalysis processes emerged in the 1970s, through studies of photoelectrochemical cells, and aimed at producing fuel with low-cost materials [40]. Fujishima and Honda in 1972 [40] described the oxidation of water containing TiO_2_ in suspension, irradiated in a photoelectrochemical cell, which promoted the generation of hydrogen and oxygen. With the advent of nanotechnology and consequent advances in the development of new functional and environmentally friendly semiconductor materials, as well as of new techniques for characterizing and testing these devices, photocatalysis processes gained prominence as a viable alternative for applications in the domain of environmental decontamination, with high efficiency and reproducibility.

In the case of metal oxides-based systems, photocatalysis is established by a natural or artificial light stimulus. The HO^•^ and O_2_^•−^ species are photogenerated from the water molecules on the metal oxide surface and promote redox reactions capable of degrading contaminant molecules [41].

**Figure 2:**
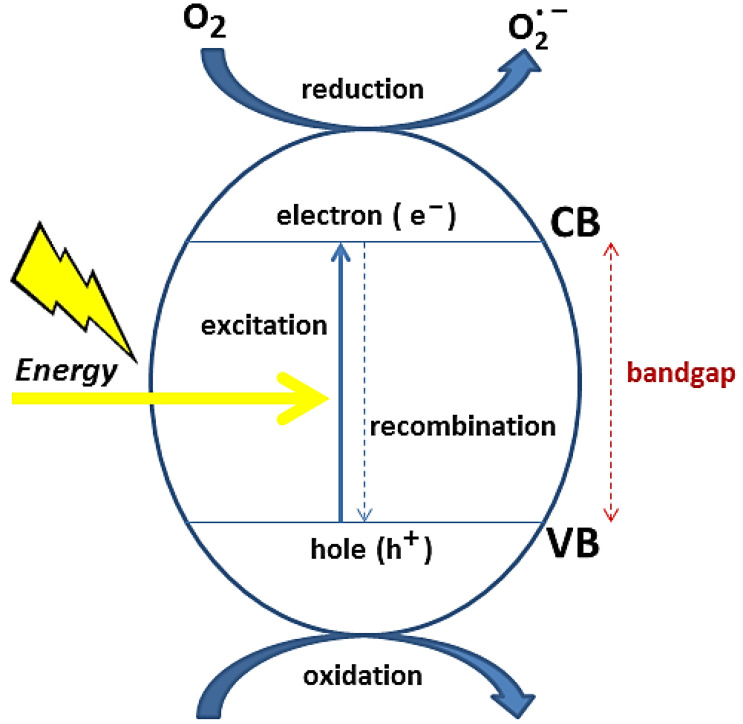
Illustration of a metal oxide-based heterogeneous photocatalysis.

The action of a metal oxide-based photocatalyst in an aqueous medium can be summarized as follows. First, the semiconductor is exposed to a light source, with enough energy to activate it: therefore, photons with energy greater than the energy of the bandgap are absorbed. This process causes an electron (e^−^) to be transferred from the valence band to the conduction band, leaving a hole (h^+^) in the valence band. Electrons (e^−^) in CB react with the oxygen of the adsorbed water molecules at the oxide surface, forming superoxide ($O_{2}^{-}$) and/or hydroperoxide (${OH}^{-}$) free radicals. Simultaneously, holes (h^+^) generate HO free radicals from the adsorbed water at the metal oxide surface [41]. An illustrative diagram of this process is represented in Figure 2.

The combined action of direct redox processes, induced by holes and photogenerated electrons on the surface of the catalysts, and of indirect redox processes, derived from reactive oxygen species, is responsible for pollutant degradation [13,42,43].

In short, the competition between the capacity for interfacial charge transfer and the recombination of the e^−^/h^+^ pairs, is a determinant factor for the photocatalytic efficiency of the materials. Basically, the action of free radicals leads to the degradation of contaminants in the form of carbon dioxide, H_2_O molecules and salts of inorganic origin.

The main challenges of achieving photocatalysis with metal oxides are their high bandgap energies needed to overcome their recombination levels of electron–hole pairs and their chemical and structural stability. Several researchers have focused their work on the synthesis of narrow-band photocatalysts, which can be used under visible light irradiation. Various surface enhancement methods to achieve this include doping with metal/non-metal ions, surface interaction with other semiconductors, or the use of hybrid nanostructures with other functional semiconductors [44,45,46].

The modification of the catalyst surface to restrict charge recombination and increase the visible light response through metal/non-metal doping has been extensively studied [47], as discussed in the previous sections. As mentioned earlier, the doping of the semiconductor catalyst with metal ions can result in the formation of intermediate energy levels in the bandgap region and improve the separation of charge carriers [48]. Between other advantages, the one that involves controlling the size of crystallites and of the surface area/volume ratio of the nanostructures must be referenced, once it will increase the adsorption of pollutants on their surface [49].

In terms of photocatalytic performance and stability of the semiconductor materials, nanocomposites based on titanium dioxide (TiO_2_), zinc oxide (ZnO), vanadium pentoxide (V_2_O_5_), niobium pentoxide (Nb_2_O_5_) and tungsten trioxide (WO_3_) stand out [50]. Metal oxide nanocomposites based on those oxides are widely studied for the reduction of organic pollutants due to their non-toxicity, high resistance to photo corrosion and availability in different crystalline forms with high photocatalytic activity [51].

The applicability of hybrid nanocomposites based on TiO_2_ anatase (which can result in materials with higher photoactivity and lower recombination rates) for removing contaminants has gained popularity due to their cost-effectiveness, allied with their surface properties, including morphology, surface area and adsorption affinity, and the possibility of absorbing visible light [52,53,54,55].

On the other hand, the recovery and reuse of these nanocomposites in new decontamination processes may represent a limitation, especially regarding the separation of nanoparticles of the reaction medium after the end of the reaction. In this sense, different strategies have been applied to overcome these problems, such as the use of membranes able to support these metal oxide nanocomposites [13].

#### Charge Transfer in MON-Based Photocatalysts by Surface Tailoring

For effective photocatalysis, the photocatalyst conclusively delivers charge carriers to the interface of the semiconductor electrolyte [56,57,58,59,60,61]. Recently, some authors have explored surface interactions to improve photocatalytic activity, for instance, using nanomaterials with tailored structure that allows one to control the orientation of certain surfaces [62,63,64].

Simultaneously, characterization techniques allowed one to look and understand more deeply the atomic structure of surfaces and their interaction with the environment [65,66]. In consequence, it was possible to shape materials with metastable surface phases that possess enhanced electronic properties, while simultaneously fine tuning their photocatalytic reactivity. Evidence of surface orientation-dependent photocatalytic activity has been reported in literature. For instance, using polycrystalline thin films of rutile TiO_2_, the photocatalytic reduction of Ag+ ions was investigated and a stronger photocatalytic activity of the {101}/{011} orientation of rutile TiO_2_ was observed [67].

Diverse mechanisms may contribute to the found surface reliance with photocatalytic reactivity. For example, if electrons and holes get diffused along different directions, diverse oxidation or reduction reactions will be exhibited by different surfaces of the crystalline structure. These differences can be due to the band structure, to polarization mechanisms, or to induced superficial electrostatic potentials caused by adsorption reactions.

Regarding band structure influence, which has its origin in the bulk properties of the materials, it was first proposed by Giocondi et al. [68] that the differences in the photocatalytic activity on (110) and (100) surfaces of strontium titanate (SrTiO_3_) was due to the bulk band structure, which caused anisotropic diffusion of photoexcited charge carriers. By close inspection of the band structure, the authors found that photoexcited electrons and holes had a momentum-vector in the h100i direction: consequently, excited charge carriers of the bulk get diffused mainly along the {100} surface, a fact used to explain the higher photocatalytic activity of this orientation.

As reported, controlling charge transport within the bulk can give rise to enhanced surface photocatalytic reactivity. However, as proposed for SrTiO_3_, the diffusion of carriers through specific surfaces may not be just due to the band structure, but may also be the result of dipoles formation, inducing holes and electrons that diffuse in opposite directions.

In unpolarized ferroelectric materials, containing several domains with opposite polarity, but of which the total net polarization vector is zero, the photocatalytic reactivity of metallic salts was used to evaluate spatial distribution of photo-oxidation/-reduction reactions. It was shown that products originating from those reactions decorate the domain structure of some ferroelectric materials, such as BaTiO_3_, after photo stimulation [69,70]: it was observed that while photogenerated electrons led to the reduction of Ag+ ions with consequent distribution along the active surface of Ag clusters, photogenerated holes conducted to the oxidation and to the subsequent decoration of the active surface with lead oxide. Thus, electrons and holes diffusion is controlled by bulk polarization and transported along domains with opposing directions. By conducting and AFM analysis, Kalinin et al. [71] defined domain patterns into the ferroelectric material, which subsequently was decorated by photoreaction resulting products.

Band bending in the near surface region can also promote direct charge transport. Photoexcitation causes electrons/holes to diffuse to the surface, promoting a down-ward/up-ward band bending near it. The generated electrical potential in the charged region enhances electron–hole separation with a consequent increase of the photocatalytic reactivity. Synthesizing, band bending is due to the created surface charges, induced by adsorption processes. Bhardwaj et al. [72], when studying the photocatalytic activity of barium strontium titanate (Ba1-xSrxTiO_3_), found a maximum in the activity for a tetragonal-to-cubic phase transition composition: they associated it with the anomalously high dielectric constant for this transition. If for different surface orientations the band bending varied, then strongly surface-dependent photocatalytic activities are generated.

Most photocatalytic reactions are carried in aqueous solutions. At the interface layer between the solution and the crystal, a charged double layer is formed, consisting of surface charges with a certain polarity, on the crystal side, and of ions with opposite polarity in the layer of the solution closest to the surface. This originates a potential barrier near the interface, which can be regulated by balancing the Fermi level of the immersed photocatalyst with the one of the electrolytes. Thus, it can be stated that photocatalytic activity depends on the potential barrier height: indeed, the height and width of the space-charged layer, which are determined by the potential barrier height, condition the electron–hole separation and diffusion to the surface. It must be pointed out that the potential drop in the interfacial layer affects ion transport along the solution into the material surface and thus, affects the photo-oxidation/-reduction of the ions at the surface. Bullard et al. [73] studied and determined the pH value at which the surface carries no net charge for different rutile single crystals. They measured force–distance curves employing an atomic force microscope and used them to determine the pH values for which the curves shifted from attractive to repulsive. Strong surface-dependent pH-values were found for the studied activated surfaces.

In the literature, it was shown that the surface electronic properties of some photocatalysts based on transition metals are quite different from the ones found for the bulk. For instance, for the rutile TiO_2_ (011) surface, a new metastable surface phase, not the typical shaped 2 × 1 one, was produced (it was experimentally confirmed that, under vacuum, one of the most common surfaces for rutile crystal, the (011) one, gets reconstructed into a 2 × 1 structure [74]). This newfound phase, which displays a much smaller bandgap than that of bulk [75], was characterized by scanning tunneling microscopy and spectroscopy (STM/STS) and photoemission spectroscopy. Indeed, it was found that the bandgap is only of around 2 eV, and that the majority of the bandgap narrowing was caused by the induction of new states on the top of the valence band of the bulk. This surface will attract holes, and consequently, it is expected that it will turn highly active regarding photo-oxidation reactions: as a consequence, electrons can transfer to other active surfaces, of the same particle, and make them available for photoreduction reactions. In summary, if the orientation of one surface can be modified by molding its bandgap, electron–hole separation processes would be boosted, and separate oxidation and reduction reactions would growth.

In a work by Lahiri et al. [76], the growth of a monolayer of zinc sulfide (ZnS) over ZnO was used to enhance ZnO photocatalytic activity. Even if ZnO and ZnS both display a wide bandgap, the presence of a ZnS monolayer induces states on top of the ZnO valence band with a consequent upward band bending [77]: similarly, as described for the new surface phase on TiO_2_ (011) [75], the formation of a similar hole-trapping layer is found for ZnO.

The phenomenon has also been observed in quantum dots/metal oxide-based nanocomposites, for the removal of toxic organic pollutants. Quantum dots (QD) are known as zero-dimensional (0-D) nanomaterials and have the peculiarity of having a very high surface area/bulk ratio. Photocatalysis processes with these materials can be improved in relation to the use of larger particles, since the electron–hole recombination is significantly reduced due to the short path for charge transfers from the crystal bulk interface to the surface of the nanocomposite [64].

In summary, strategies that allow modifying surface electronic conduction characteristics will enhance not only the materials’ bulk properties but also the creation of charge-trapping layers at the materials surface.

## 3. Bibliometric Study

As a brief characterization of the research field on metal oxides nanocomposites for remediation of aqueous pollutants, a data search was performed in the Scopus platform (April 2023). The search was carried out using “metal oxide”, “composite” and “photocatalysis”, as keywords for their presence in the title, abstract or keywords of the documents. This search resulted in 563 hits from 2012 up to the current date. The collected data were organized and analyzed in the following ways: (i) trends in publications over the years; (ii) main journals, their Impact Factor and CiteScore; (iii) transitions in the keywords over the years; (iv) word-cloud concept of the main aspects related to the topic. Analyses were performed using the RStudio^®^ (version 3.5.2) and VOSviewer (version 1.6.14) software packages.

**Figure 3:**
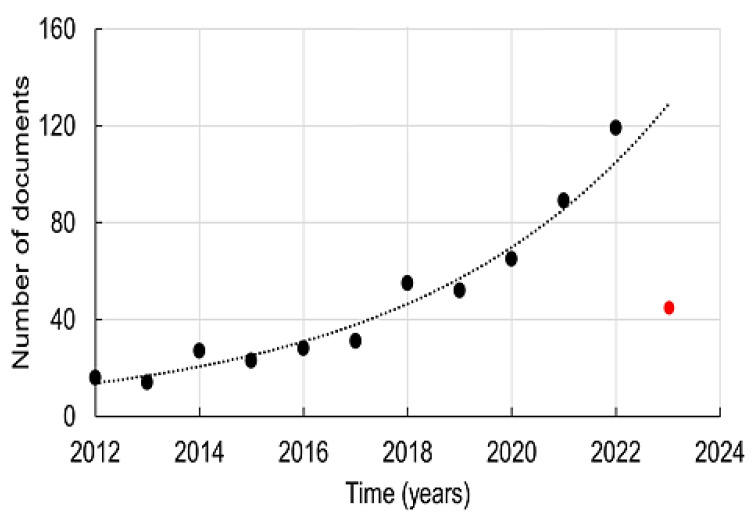
Publishing trend in the field on metal oxide nanocomposites for remediation of aqueous pollutants observed between 2012 and April 2023.

Figure 3 shows the trend regarding the quantity of documents published from 2012 up to the current date on metal oxides nanocomposites for remediation of aqueous pollutants. The first six years were a period with low variation in the number of published documents (between 14 and 31 per year).

After 2019, the number of published documents increased exponentially, reaching 119 documents last year. This growth can be linked to the need for developing water decontamination solutions, in the face of the expanding evolution of global environmental pollution. This behavior has been observed for research in functional nanomaterials, which involve a series of peculiarities, such as the discovery and synthesis of new materials, the development of more efficient characterization methods and the study of new mechanisms and collaborative interaction between selective components, which ensure greater surface area against contaminants [78].

This was found to take place with studies involving metal oxides and their nanocomposites in recent years, while looking for selective materials and synthesis techniques that guarantee a more efficient and environmentally friendly water decontamination of organic pollutants. The reduction in the number of literature reports in 2023 is related to the period under analysis, which was performed in April.

**Figure 4:**
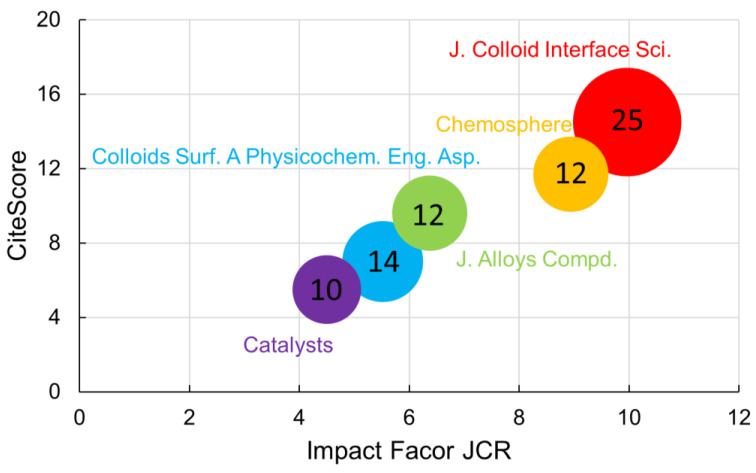
Top five scientific journals contributing to the topic of metal oxide nanocomposites for remediation of aqueous pollutants.

The top five journals with more published documents over the subject are listed in Figure 4. The ranking is led by the Journal of Colloid and Interface Science (25 documents), followed by Colloids and Surfaces A: Physicochemical and Engineering Aspects (14 documents) and by Chemosphere (12).

Much of the research on metal oxide nanocomposites for the remediation of aqueous environments is distributed through numerous papers in the materials domain that deal specifically with the synthesis of new materials, their characterization and their application in solving current problems in society. The main journal where more reports on the use of metal oxides for remediation of aqueous pollutants were published is also the journal with the highest Impact Factor and CiteScore, once it connects the study of basic science of functional materials with the discussion of new and (or) improved techniques to remedy environmental pollution by hazardous materials, such as organics and heavy metals.

**Figure 5:**
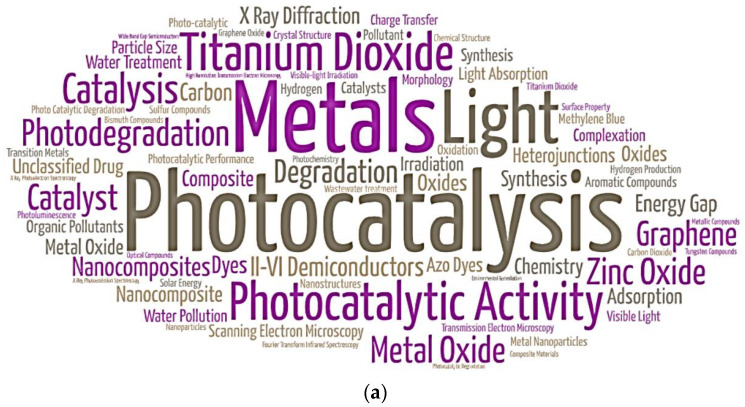

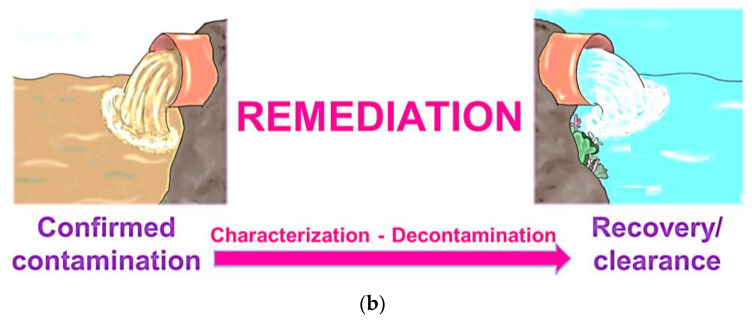
(**a**) Word cloud of the main keywords that appear in documents of metal oxide nanocomposites for remediation of aqueous pollutants. (**b**) Illustration of standard water remediation flow of activities.

A word cloud illustration is presented in Figure 5a. It shows the most frequent keywords that emerge in the documents that involve metal oxide nanocomposites for remediation of aqueous pollutants. ‘Photocatalysis’ (414), ‘Metals’ (155), ‘Photocatalytic Activity’ (146) and ‘Titanium Dioxide’ (121) were the keywords with the highest number of results in the performed search.

Water pollutants photocatalysis shows itself as one of the most used and promising techniques in comparison to conventional water decontamination techniques, mainly due to its environmentally friendly use and constant development of materials with better surface properties. In addition, it has been demonstrated that aqueous environment pH level is one of the main factors to be considered, because the activation of surface functional groups of potential nanocomposites highly depends on it. In the same figure, Figure 5a, it is possible to find words, such as ‘ferric compounds’, ‘iron’, ‘iron oxide’ and ‘magnetism’, which refer to the recent use of metal oxide nanocomposites based on ferroelectric materials, in order to improve the efficiency of photocatalysis and adsorption processes, and consequently, also in the separation of contaminants from water.

High impact water pollutants, such as pharmaceuticals, pesticides, organic dyes and heavy metals, have been widely cited in the search because they represent a large portion of the substances used in industrial processes, thus showing that the search for suitable clean-up methods is an open field of research. In general, the analyzed works discuss the stages of the decontamination, highlighting the standard remediation flow of activities, shown in Figure 5b, composed of confirmation of contamination, characterization of the contaminant, decontamination process and recovery/reuse.

**Figure 6:**
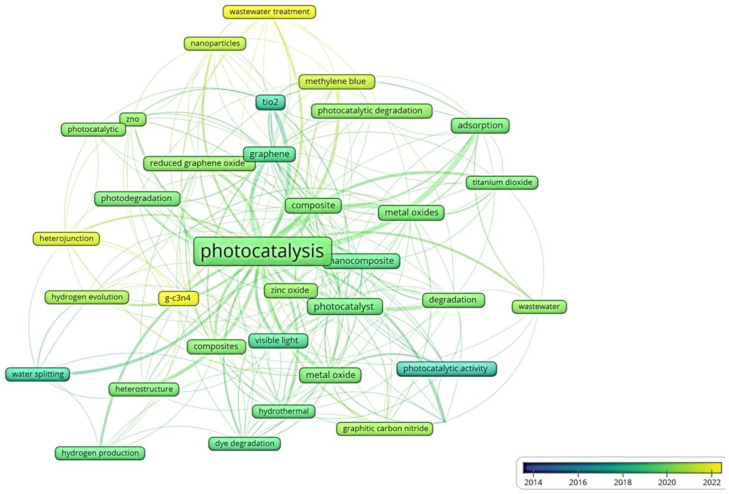
Illustration of keywords—transition in the representative documents of the use of metal oxide nanocomposites for remediation of aqueous pollutants.

An analysis was performed of the keyword’s transitions over the last nine years, which appear at least 10 times in the text of the found documents in the scientific databases, which can be seen in Figure 6: it illustrates an interest shift in the research field, which stands for the advances in the use of metal oxide nanocomposites for the remediation of aqueous pollutants.

The analysis shows that in the most recent years, mixed MO and carbon materials have been highlighted for the production of MON used in photocatalysis processes. The excellent photodegradation results obtained using these materials have been attributed to the chemical synergy between the components in the nanocomposite, to the greater efficiency in charge transfer and to the reactive surface areas (much larger than those observed for pristine materials). The analysis also showed that graphitic carbon nitride (g-C_3_N_4_) emerges as a new functional material that can interact with metal oxides and is used to produce more efficient photocatalyst devices [78,79]. The pollutant-specific search revealed a huge interest in remediation of dyes and wastewater in general.

In the examined searches, the study of mixed MO-based nanocomposites for remediation of aqueous pollutants was frequent. Titanium dioxide in its allotropic anatase form (anatase TiO_2_, ~3.2 eV bandgap) has been the most used metal oxide for the production of these nanocomposites, which when synthesized with other oxides or with other functional materials, allowed one to improve photocatalysis processes [52,53,54,55]. This oxide is non-toxic and possesses high chemical stability, excellent photoactivity and photostability, easy UV-activation, and low cost when compared to other oxides. The anatase phase is preferable to the rutile one (irreversible transition at 600–700 °C), mainly because it presents a greater reactive surface area, for interaction with other materials [55].

Regarding the use of carbon nanostructures for the photodegradation of organics, graphene stands out. In the last decade, graphene has gained very high attention from both the scientific and industry communities, due to its unique physicochemical, electrical and optical properties, all combined with its honeycomb structure.

These materials ensure high surface area and a wide variety of functional groups, which can be used for several emerging applications, such as water decontamination. On the other hand, their potential strongly depends on the choice of a suitable synthesis technique. Based on the discussed aspects, the next section highlights recent advances on the use of mixed MO, conducting polymers and carbon materials in MON photocatalysts.

## 4. Recent Advances on the Use of Metal Oxide-Based Nanocomposites (MON) in Photocatalysis of Organics

### 4.1. Mixed Metal Oxide-Based MON

Efforts have been applied to develop synthesizing methods that make it possible to obtain metal oxide (MO)-based photocatalysts with controlled size, morphology and crystalline phases. Among them, the hydrolytic sol-gel, oxidation, sputtering, epitaxy, hydrothermal, solvothermal and sintering approaches stand out [80].

With these methods, it is possible to produce mixed MO nanocomposites composed of two or more metal oxides which interact at the surface and/or atomic level [81]. They have been preferentially used in technological applications, such as photocatalysts, due to the possibility of displaying improved structural and electronic properties, when compared to those known for their constituent oxides used individually [82]. Surface interaction between the oxides is possible, which results in a material with crystalline phases of the precursor semiconductors, but without the formation of solid solutions. When the interaction takes place at the atomic level, with two or more metallic cations involved, adding to the possibility of formation solid oxide solutions (such as perovskites, spinel, scheelite and palmeirite), there is the chance of vacancies from the host oxide to become occupied by dopant metal atoms [83,84]. These interactions provide superior chemical reactivity for decontamination of aquatic environments by photocatalysis.

The interactions between the constituent metal oxides of the nanocomposite materials have been one of the most explored approaches used in the study of enhanced photocatalytic properties of these nanostructures. The increase in photocatalytic activity stems from a combination of improved photo response, charge separation efficiency and the presence of multiple active sites on the hybrid nanostructures. Generally, these heteronanostructures of mixed MO can vary, for example, from binary, ternary to quaternary nanostructures [85,86,87,88,89,90,91,92,93,94].

**Figure 7:**
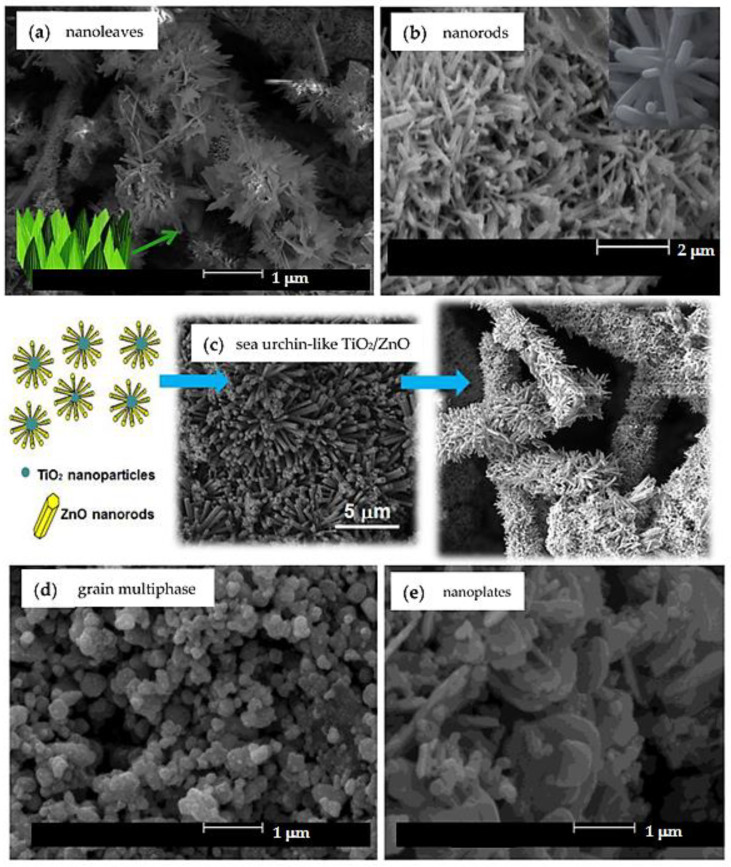
(**a**) SEM images of nanoleaves- and (**b**) nanorods-like 3D hierarchical nanostructures; (**c**) scheme and micrograph of sea urchin-like TiO_2_/ZnO hierarchical nanostructure (**left**) and polymeric fibers radially decorated with this MON (**right**); (**d**) grain multiphase and (**e**) plate-like heterogeneous nanostructures. Source: Authors’ files.

Among the studied mixed MO nanostructures, 3D hierarchical and heterogeneous ones stand out (Figure 7). Three-dimensional hierarchical nanostructures have well-defined and organized shapes when compared to other types of nanostructures. Nanotrees, nanoleaves (Figure 7a), nanorods (Figure 7b) and sea urchin-like structures (Figure 7c) are some examples of these nanostructures that have been used in photocatalysis applications [85,86,87,89,94].

TiO_2_/ZnO hierarchical heteronanostructures are potential materials for photocatalysis processes against organic dyes [95]. The sea urchin-like TiO_2_/ZnO nanostructures (see Figure 7c) can be produced by hydrothermal growth of zinc oxide hexagonal nanorods on the surface of P25 TiO_2_ (3:1 anatase:rutile ratio) nanoparticles: it shows high surface area to dye photodegradation, even with white light (which does not occur with the use of pure TiO_2_) [95]. The best decontamination results obtained with TiO_2_/ZnO hierarchical nanostructures have been attributed to the atomic and surface interactions between the oxides. This configuration reduces the bandgap of the combined material and, consequently, hinders the e^−^/h^+^ recombination. Later, the authors proposed the radial growth of zinc crystals on the poly(methacrylic acid-co-ethyl acrylate) nanofibers surface, prepared by electrospinning and decorated with TiO_2_ particles: with this procedure, photocatalysts with high surface area/volume ratio and better performance, 90% of dye degradation in aqueous solution after 70 min of exposure to light, when compared to that obtained using the sea urchin-like nanoparticles, were developed. In this last configuration (see Figure 7c, right), the fibers functioned as a polymeric template to stimulate the regular distribution of oxides and prevented particles aggregation, which significantly increased the process efficiency.

In this sense, Bao et al. [96] developed a novel sea urchin-like SiO_2_/TiO_2_ hierarchical nanocomposite, which degraded 82.2% of the RhB dye under UV light, in 35 min. The higher photocatalytic activity was attributed to the core–shell configuration of the nanocomposite, which ensured efficient charge transfer for the formation of selective dye-degrading radical species.

When talking about heterogeneous (grain multiphase (Figure 7d), nanoplates (Figure 7e), among others) MO nanostructures [81,97,98,99,100], it is necessary to understand that the interaction between two or more metal oxides is seen as a viable alternative for the fabrication of MON systems with looked-for properties by simpler synthesis methods than those used for 3D hierarchical ones. The chosen synthesis method results in different textural, optical and morphological properties of the photocatalysts, which directly influence their chemical stability and recyclability [93,101]. Chemical and physical parameters such as precursors, solvents, pH, pressure and temperature must be considered during the synthesis process of these nanocomposites [99].

Advances in this field of research involve the incorporation of zinc (Zn), tungsten (W), vanadium (V), niobium (Nb), indium (In), iron (Fe), nickel (Ni) and copper (Cu) oxides into TiO_2_ [102]. Zinc oxide (ZnO) is widely used in photocatalysis processes due to its physical–chemical characteristics (biocompatible, non-toxic, chemically and mechanically stable, insoluble in aqueous environments, ~3.1–3.3 bandgap energy, and so on), similar to those of anatase TiO_2_. Nanocomposites based on TiO_2_ and ZnO, produced by various methods, have shown better photocatalytic properties when compared to those obtained for single pristine TiO_2_ [103,104,105,106].

Hybrid TiO_2_ nanoparticles have been synthesized by hydrothermal processes, with the incorporation of zinc oxide in the atomic structure (substitution of Ti^4+^ by Zn^2+^) and on the surface of TiO_2_ [107]. TiO_2_/ZnO grain multiphase heterogeneous nanostructures are known to accelerate the degradation process of dyes under visible light, with a mutual chemical process of N-deethylation and cleavage of contaminant molecules [107,108]. Electrons transfer from the zinc oxide conduction band to the titanium dioxide conduction band is described by the bands theory. Consequently, there is photogeneration of holes (h^+^) from the TiO_2_ valence band to the ZnO valence band. With this configuration, and based on the standard photocatalysis mechanisms, the separation of electrical charges is said to occur in an improved way [107,108].

For instance, the interaction of tungsten trioxide (WO_3_) with TiO_2_, obtained through a sintering process, increased the conductivity of the TiO_2_/WO_3_ system in comparison to the observed for pure TiO_2_ [13], while doping TiO_2_ with niobium pentoxide (Nb_2_O_5_) [109] or vanadium pentoxide (V_2_O_5_) [110] delays the phase transition from anatase to rutile.

**Figure 8:**
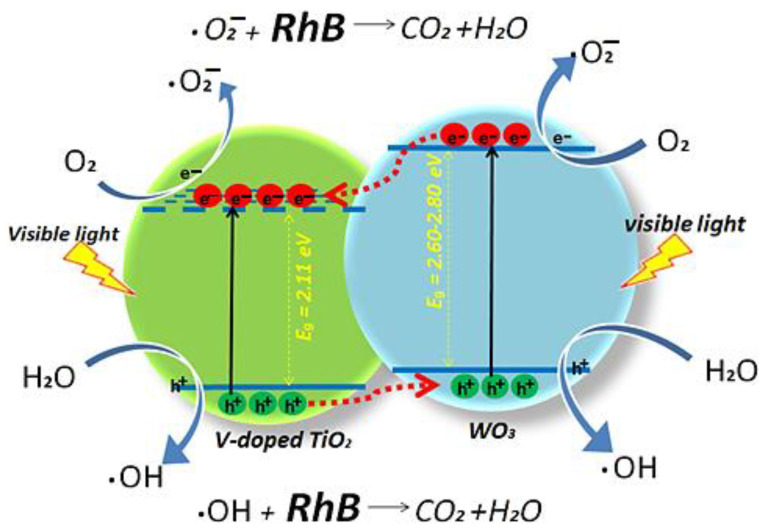
Ilustration of the RhB dye photodegradation mechanism using a V^5+^-doped TiO_2_/WO_3_ MON.

In addition, Pereira et al. [13] showed that the V^5+^-doped TiO_2_/WO_3_ granular three-phase nanocomposite, produced by a simple calcination process at 500 °C, and then supported on electrospun polymeric membrane, has a superior performance regarding the photodegradation of RhB under visible light, in relation to the use of anatase TiO_2_, the anatase TiO_2_/monoclinic WO_3_ binary system, and the same ternary mixed MO in powder form (support-free). TiO_2_ doping using 5 wt% of V_2_O_5_ in a 1:1 TiO_2_/WO_3_ mixture (in mol) considerably reduced the bandgap of the first oxide from 3.2 eV to 2.11 eV. The dye photodegradation mechanism is illustrated in Figure 8.

Furthermore, the immobilization of the ternary grains on the polymeric membrane prevented the particles aggregation and promoted a high surface area of action for the nanocomposite. The combination of these characteristics allowed the novel photocatalyst device to degrade up to 90% of RbB dye after 120 min of exposure to a 60W white light source. The use of the membrane also facilitated the removal of the photocatalyst from the reactor environment for its reuse in up to three reuse cycles, without loss of process efficiency.

Other nanomaterials such as Cu/In/Ti [111], Ni/Co/Fe [112], G/Fe/Ti [113] and Zn/Ni/Al [114] ternary mixed MO, also showed photocatalytic properties superior to those of TiO_2_ regarding the degradation of the usual organic dyes.

Researchers have also been focusing their research on mixed MO nanostructures for the treatment of wastewater systems contaminated with pharmaceuticals and agrochemical products [93,102]. For example, the production and use of antibiotics and pesticides has increased in recent years, with as a direct consequence f the inappropriate discard of these products in the environment [93,115,116,117,118,119,120].

Plate-like Zn/Fe mixed MO nanostructures, synthesized by calcination of their precursors at 300 °C, promoted the simultaneous removal of several high-demand pharmaceuticals (ibuprofen, acetaminophen and diclofenac) by photodegradation [121]. No drugs were detected in the solution after 12 h of exposure to the light source, in each of the six usage cycles. The photodegradation mechanisms were discussed, and h^+^ reactive species were considered as primarily responsible for the breakdown of the organic pollutant molecules. In addition, the photocatalysis process under simulated solar irradiation was found to be of high efficiency and low cost.

Doan et al. [115] developed a novel grain multiphase Cu_2_O/Fe_3_O_4_ heterogeneous nanostructure supported on Fe metal–organic frameworks (MOFs) as an efficient photocatalyst for the degradation of ciprofloxacin (one of the most widely used broad-spectrum antibiotics to treat serious infections) under visible light. The authors showed that the immobilization of the oxides in the MOFs returned a nanocomposite with characteristics of minimal aggregation, large surface area and porosity, which guaranteed 99.2% of degradation of the antibiotic at pH of 7, in up to 105 min of exposure to light, reusable in five cycles of decontamination.

More recently, the work by Ayah et al. [93] showed that, using mixed MO-based MON, it is possible to degrade 98% of tetracycline antibiotic molecules (frequently found in the wastewater of animal husbandry, hospitals, and pharmaceutical industries) in a 20 mg/L contaminated aqueous solution, within 105 min. The authors used mixed nanostructures based on iron oxide and bismuth tungstate (Fe_3_O_4_/Bi_2_WO_6_) supported on g-C_3_N_4_ nanosheets, which ensured a bandgap of 1.3 eV, much lower than that of pristine oxides (2.9 eV) and of g-C_3_N_4_ (2.7 eV). This configuration guaranteed six cycles of reuse of the material, inhibited the negative impact of oxide particle agglomeration and significantly decreased e^−^/h^+^ recombination of the photocatalysis process under visible light, highlighting the effectiveness of HO^•^ and O_2_^•−^ reactive oxidants generated by the tetracycline degradation.

Farrukh’s Group [117] has synthesized cerium (IV) oxide/silicon dioxide (CeO_2_/SiO_2_) heterogeneous grains by surfactant assisted via sol-gel process for the photodegradation of Chlorpyrifos (one of the most widely used insecticides in the world, but which can cause many genotoxic and neurological damage, especially in children). The results showed that the nanocomposite is capable of degrading 90% of the pesticide in 150 min using UV light.

In the search for new alternatives to circumvent this problem, Saljooqi et al. [118] developed a mixed MO for degradation of this insecticide under visible light with superior performance. The group showed that the Fe_3_O_4_/TiO_2_ nanocomposite uniformly distributed on a porous ZnO structure is capable of degrading 95% of Chlorpyrifos, for an initial concentration of the pollutant about eight times greater than that used in the first case, and in a much shorter time period (50 min). The enhanced photocatalysis results were attributed to the selective oxides heterojunction, which promoted excellent spatial separation of charge carriers.

Kumari et al. [122] synthesized a hierarchical ZnO/CuO nanocomposite by hydrothermal process for the treatment of Triclopyr-contaminated water (a widespread herbicidal agent for post-emergence control of weeds in pastures). The pesticide contained in a 10 mg/L (pH 4) contaminant aqueous solution was completely degraded after 100 min of exposure to UV light. Mg/Al, Mg/Cerium (Ce) binary metal oxides are also seen as potential materials for the degradation of pesticides because they offer quicker, efficient and economically viable solutions for the photocatalysis process [123].

In general, interactions at the atomic level and at the interface between the oxides return nanocomposites, with a higher surface area, improved photocatalysis results in water cleaning processes [102]. Table 1 shows some of the most recent research works on the use of mixed MO for the degradation of antibiotics and pesticides in water.

### 4.2. (MO/Conducting Polymer)-Based MON

Other possible ways to produce water-decontaminating materials include the interaction between metal oxides and conducting polymers. Conducting polymers (CPs) are functional organic materials that stand out from other polymers because they display electrical conductivity. Polyaniline (PANI), polypyrrole (PPy), polythiophene (PT), polyacetylene (PA) and poly (3,4-ethylenedioxythiophene) (PEDOT) are some examples of CPs explored in the literature [140].

The intrinsic electrical conduction is attributed to the specific chemical structure of these polymers, with alternating single and double bonds (which gives them the classification of conjugated polymers). These semiconductors have HOMO (highest occupied molecular orbital) and LUMO (lowest unoccupied molecular orbital) layers, which levels are similar to those observed for the VB and CB in metal oxides, respectively. As in organic semiconductors, there is an energy gap between HOMO and LUMO orbitals that defines the electronic properties of the material. When the CP molecules are photoexcited (polarons), electrons from the HOMO layer migrate to the LUMO layer, leaving a hole in the HOMO layer. This molecule will be called polaron-hole or polaron-electron, under oxidation or reduction, respectively.

Conducting polymers are also known for their unique microstructural (high porous bulk and high surface roughness, high surface area/volume ratio and a large variety of functional surface groups), for their processability by dispersion, for their adjustable oxidation-reduction and electrical/electronic properties by organic synthesis from their monomers, and for their dispersion abilities of other electronically active materials, such as metal oxides [141]. They also distinguish themselves due to the possibility of including natural clays, with a consequent increase of the chemical and/or structural stability of the polymeric matrix.

In addition to the chemical functionalities of their polymeric matrix, CPs are used as templates for the dispersion of metal oxides, in which they have shown a capacity for preventing the aggregation of the particles. Thus, the combined action between oxides and polymers significantly increases the surface area of action with contaminants.

The properties of the combination of CPs with metal oxide gave origin to a variety of applications in nanotechnology, such as in the development of sensors, supercapacitors, solar cells, organic electronic devices and new efficient photocatalysts materials for the remediation of aqueous environments polluted by organics [142].

Regarding the photocatalysis mechanism using (MO/CP)-based MON, the conducting polymer can act as a visible photosensitizer, exciting electrons from the HOMO to the LUMO. In sequence, these electrons are then collected in the metal oxide CB, making them available for the degradation processes. In addition, there is an interaction between CP HOMO and metal oxide VB, with transfer of holes from the oxide VB to the polymer HOMO layer. The higher density of electrons and holes available in MON are the main causes of the better photodegradation performance of organics compared to the use of pristine MO [143,144].

More than 80% of the research papers (returned by bibliometric analysis) on CPs containing MON for application in photocatalysis of organics use the PANI and PPy polymers, largely due to their being the first CPs discovered, with properties widely disseminated in the literature and viable synthesis routes with a low production cost when compared to newer CPs. PANI is a conducting polymer known to have a large amount of amine and emine superficial functional groups. These groups have a strong affinity with metal ions and can therefore be used to remove selective organics in aqueous systems. The research field concerning the development of metal oxide–PANI nanocomposites is open and has allowed many innovations along recent years. For example, the ZnO-PANI nanocomposite was recently introduced as a new photocatalyst for the removal of Acid Orange 8 dye in aqueous solutions [145]. The interaction of zinc nanostructures with PANI let one obtain greater mechanical and thermal resistance, and to improve the adsorbent properties of the OH functional groups concerning oxide and amine groups of polyaniline (available in large quantities and with high dispersion).

The iron oxide–PANI core–shell nanocomposite was prepared using surfactant-assisted sol-gel and polymerization routes [146]. The nanocomposite showed very high degradation efficiency against methyl blue (99.8), eosin yellow (98.5%) and methyl red (99.6%) organic dyes, with four recyclability cycles along 50 min. The best results were linked to the PANI surface protection against Fe_2_O_3_ corrosion, to the low e-/h+ recombination rates and to the nanocomposite’s broad ability to generate reactive oxidative species in water, which accelerated the reaction of dyes degradation and substantially increased the process efficiency.

In addition, the arrangement of a ternary system, with two selective oxides and PANI, can considerably increase the mobility of charge carriers and, consequently, improve the photocatalysis performance against organic contaminants in water [147]. TiO_2_-CoFe_2_O_4_ [148], TiO_2-_ZnFe_2_O_4_ [149], rGO-ZnFe_2_O_4_ [150] and rGO-MnO_2_ [151] have been successfully combined with PANI to obtain new photocatalysts with better performance. Recently, the TiO_2_/Bi_2_O_3_/PANI ternary nanocomposite was synthesized as a novel photocatalyst material [152]. The results were very promising, with photocatalysis abilities against RhB dye (100% degradation in 40 min) and Triclopyr pesticide (85% degradation in 120 min). The photocatalysis process results established this (MO/CP)-based MON configuration as a sustainable material that can be reused repeatedly.

Polypyrrole (PPy) is another conducting polymer with great potential to form functional nanocomposites with metal oxides. PPy is synthesized after polymerization of the pyrrole and activated as a conductive polymer by means of an oxidation process. This polymer is amorphous and has a fractal surface, which guarantees a high surface area in relation to its volume. Its electrical conduction properties are dependent on the conditions and reagents used in the synthesis [153,154].

TiO_2_-SiO_2_/PPy [155,156], tin dioxide (SnO_2_)/PPy [157,158], ZnO/PPy [159,160,161] have also been used for the photocatalysis of organics in water under visible light. The results showed that these nanocomposites have great affinity with contaminant molecules and new sensory characteristics. The polymerization process, morphology, stability of nanoparticles in solution and adsorption capacity characteristics are directly related to the better photocatalytic results of these nanocomposites.

Recently, Biju et al. [162] developed a photocatalyst (CuO-ZnO)/PPy that enhanced photocatalytic degradation of methane yellow (a dye widely used in the world in industries like textiles, food, cosmetics, among others) under visible light. The incorporation of PPy resulted in a 50% increase in performance in comparison to the use of the binary mixture of oxides, with greater efficiency and economy. Photocatalysis was governed by the standard mechanism previously described for MO/CP nanocomposites.

### 4.3. (MO/Carbon Materials)-Based MON

Other metal oxide nanocomposites with a high potential of usage in the decontamination of aqueous environments are those prepared by interaction with carbon materials, such as graphene (G), single- and multi-walled carbon nanotubes (CNT) (namely, SWCNTs and MWCNTs, respectively), fullerenes, among others [163,164,165].

The interaction between metal oxides and these carbon materials permits one to synthesize nanocomposites with an active surface area greater than their pristine components, and with chemical interactions due to van der Waals forces, that guarantees them the capacity of preventing the aggregation of oxides while simultaneously increasing the photocatalysis process efficiency [166,167].

The combined use of carbon materials and metal oxides (MO), as a composite photocatalyst, is known to assure facilitated charge transfer under the action of light (by different mechanisms) and to drastically decrease the e^−^/h^+^ recombination in MON [168,169,170]. The photodegradation efficiency will be directly related to the nature of the light source (UV or visible) and with the light adsorption capacity of the pollutant.

**Figure 9:**
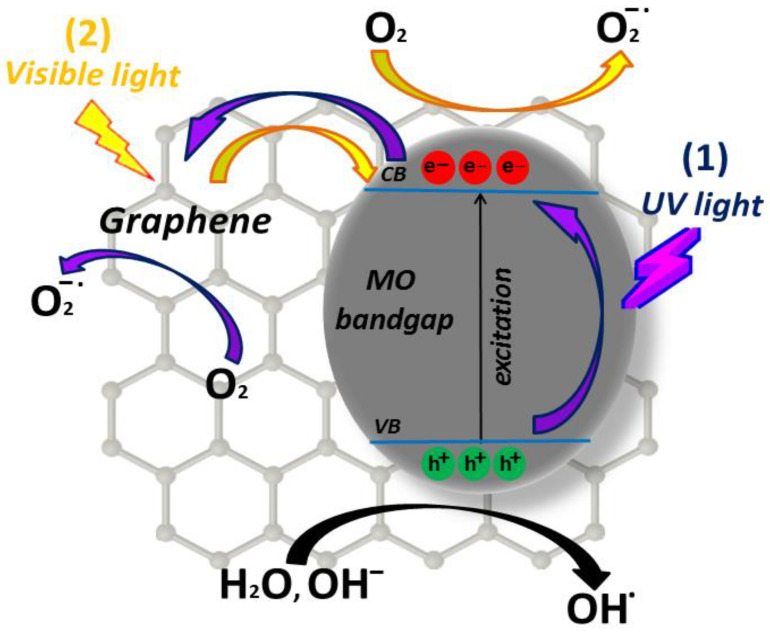
Illustration of (1) and (2) charge transfer mechanisms in (MO/G)-based MON under visible and UV light action.

For example, if the contaminated molecule is normally adsorbed on the photocatalyst surface but it does not adsorb light under UV light excitation, when photon energy of the light greater than the MO bandgap energy is supplied, primarily electrons are transferred from the BV to the BC of the metal oxide, generating a pair e^−^/h^+^ (first charge transfer mechanism). The photogenerated electrons in the MO are then injected into the carbon material because of its more positive Fermi energy [168,169]: indeed, the work function (minimum energy required to strip an electron from the material surface) of carbonaceous species generally displays more negative potentials than the MO conduction band position, which favors the process of electron ejection towards the carbon material [169,170]. In other words, the increase in MO charge separation efficiency promoted by the carbon material, combined with its excellent charge carrier mobility (which also easily sequesters electrons from available oxygen in the solution), significantly improves the performance of the MON-based photocatalyst. In addition, when MON excitation occurs with visible light, primarily a charge transfer in MON occurs via photogenerated electrons from the photoexcited state of the carbon material towards the CB of the metal oxide (second charge transfer mechanism). In this case, the presence of additional electrons in the MO promotes the reduction of its bandgap, with a consequent reduction in the e^−^/h^+^ recombination rate and in a more effective photocatalytic activity under visible light. Thus, there is a higher density of electrons in the solution that react with oxygen, available to form superoxide radicals which are responsible for the degradation of the organic material. These charge transfer mechanisms, respectively, (1) and (2), are illustrated in Figure 9 for a TiO_2_/G MON.

If the dye has a high light adsorption capacity, under UV excitation, the mechanism is the same as described for (1) in Figure 9. With the use of visible light, the dye, besides being a polluting molecule, also becomes a sensitizing agent for the transfer of electrons from its excited state to the CB of the metal oxide.

In this sense, graphene (G) can act as a photoelectron acceptor in photocatalysis processes, increasing the light absorption range (including the visible spectrum). TiO_2_/graphene nanocomposites are one of the most studied (MO/G)-based MONs for application in photocatalysis of dyes, pesticides, pharmaceuticals and sub-products of chemical industries [171,172,173,174,175]. The high capacity of graphene to accept electrons decreases the TiO_2_ bandgap, which optimizes the photocatalytic activity of the semiconductor [176]

In addition, the interaction of graphene with the copper/tin binary MO (Cu_2_O/SnO_2_) and with SnO_2_, and N-doped ZnO/graphene/ZnO sandwich composites, also results in the appearance of additional valence bands that guarantee better photodegradation results, when compared to those obtained using pristine oxides [177,178].

Regarding more recent applications, Bogale et al. [19] studied the photocatalytic degradation of methylene blue (MB) dye under visible light, using a cuprous oxide (Cu_2_O)/graphene nanocomposite. The hybrid materials were produced by a simple sol-gel method using accessible materials such as graphite powder and copper nitrate.

In summary, graphene is a viable alternative to decrease the usual electron/hole recombination in the oxides, which considerably improves the MB degradation results (94% efficiency for the Cu_2_O/graphene nanocomposite against 67% for the pure oxide, after 180 min of continuous light action).

Table 2 summarizes some recent successful case studies using MO/carbon materials-based MON for application in the remediation of contaminated water by organics, mapped in the literature search.

Other possibilities involve enhancing the photocatalytic performance of metal oxides with carbon nanotubes (CNTs). CNTs are allotropes of carbon with a cylindrical nanostructure (like a rolled graphene sheet), single- or multi-walled, named SWCNTs and MWCNTs, respectively.

In the literature, several MOs (such as TiO_2_, ZnO, CuO, WO_3_ and NiO) have been successfully incorporated into CNTs surfaces to produce potential MO/carbon-based MON photocatalysts [26,191,192,193,194]. CNTs combine adsorptive mesoporous surfaces, strong chemical durability, and high electron storage capacity, capable of providing additional photocatalysis performance, based on the acceptance of photogenerated electrons from the metal oxides by the formed MO-CNT heterojunctions.

Sapkota et al. [191] synthesized a series of CuO hierarchical nanoflowers/SWCNT heteronanostructures by simply calcining the mixture at 550 °C. With this low-cost and effective synthesis route, the authors demonstrated how to attain a degradation of 96% of the MB dye in 2 h, using visible light. Besides, the nanocomposites were used for five cycles, without loss of photocatalytic performance or morphology.

Recently, a ZnO/CNT heteronanostructure was synthesized by recrystallization followed by thermal decomposition, as another efficient and low-cost MON [193] for heterogeneous photocatalysis. This MON showed superior results regarding photodegradation of MB in water, when compared to other results reported in the literature using the same nanocomposite: previous works have shown dye degradation between 71 and 100%, with UV light or artificial white light, while the authors reached total dye degradation in 120 min, using sunlight, and for up to five cycles, which boosts the potential usage of the material in the decontamination processes. A more recent work by Hanif and collaborators [194] showed even better results using N-doped ZnO/CNTs nanocomposites, with degradation saturation attained in just 25 min. In addition to the expected interaction between ZnO and CNTs, the doping of ZnO with N decreased the MO bandgap and, thus, the transfer of charges occurs more easily, with an increase in the kinetic constant of the photodegradation reaction.

A novel TiO_2_/CNT (70/30 wt%) binary heteronanostructure was successfully synthesized by de la Flor et al. [195] as an effective sunlight photocatalyst for the degradation of a group of pesticides widely used in agriculture (thiacloprid, imidacloprid and clothianidin). With this configuration, a percentage of pesticide degradation above 80% was obtained after 180 min of photoreaction, allowing for MON reuse for up to eight cycles.

The synthesis of MON photocatalysts using fullerenes represents the most recent research topic among carbon materials reported here, proving to be a growing field. Fullerenes are an allotropic phase of carbon, with a molecular arrangement in the hollow truncated polyhedra architecture (bucky balls form), usually with 20 (C_20_), 60 (C_60_), 70 (C_70_) or more carbon atoms linked by unsaturated bonds. When they display hollow three-dimensional geometry and electronic nature structure, excellent magnetic, structural, electronic and optical properties are exhibited. These nanostructures are excellent electron acceptors, which can significantly improve electron transfer in MON [188,196,197,198].

The C_60_ (truncated icosahedron geometry) species stands out as one of the first compounds of this family, widely known and most used in heterogeneous photocatalysis processes [188]. Its combination with metal oxides has provided the synthesis of nanocomposites with different architectures such as core–shell, hierarchical and support matrix [188,189,190,196,197,198].

Zhang et al. [197] reported the easy synthesis of an efficient and reusable TiO2/fullerene photocatalyst, by mixing TiO_2_ and polycarboxylic functionalized fullerene acid through an ultrasonication/evaporation route. It was demonstrated that the introduction of C_60_ (0.5–3 wt% ratio) in the system enhanced the visible photocatalytic activity of the final material, with the simultaneous participation of photogenerated hole (h+) and superoxide radicals in the photodegradation of RhB dye. The reaction efficiency was greater than 95%, within 150 min under Xenon lamp irradiation, using at least 1 wt% of C_60_.

Munawar et al. [188] successfully synthesized a cerium (IV) oxide (CeO_2_)@C_60_ core-shell photocatalyst. The MON was able to degrade 100% of P-nitroaniline (a chemical reagent used in the preparation of several dyes) in 75 min, under sunlight. More recently, a fullerene-supported lanthanum oxide (La_2_O_3_-C_60_) nanocomposite was produced by a simple solution method and exhibited excellent organic dye photodegradation results [198]. This MON completely degraded the MB dye molecules in water, after 30 min of photoreaction under UV light irradiation, with seven usability cycles. The authors attributed the high photocatalysis efficiency of the nanocomposite to its lower bandgap and lower recombination rate of charge carriers. Other efficient MO/fullerene photocatalysts found in recent literature are shown in Table 2.

In works that address the identification of by-products of the degradation reactions, some materials characterization techniques such as liquid chromatography coupled to mass spectrometry (LC-MS) and LC-MS with mass/charge analyzer TOF (Time of Flight) (LC-MS-TOF) have been shown to be effective for the identification of the intermediate products [199,200]. However, it is important to highlight here that many research works focus on the efficiency of the decontamination process using the photocatalyst material (obtaining intermediate or complete degradation of the contaminant) and do not categorically discuss which specific by-products were formed during the reaction [201]. It is known that during the photocatalysis process, the physicochemical properties of the catalyst (which directly interfere with the reaction kinetics) promote the molecular selective modification of the contaminant, resulting in different preferential degradation [202]. The state of the art on the photodegradation of organic pollutants shows that the degradation of these molecules with heterogeneous photocatalysis processes involves some basic pathways. At first, the organics are adsorbed on the surface of the catalyst. The catalyst/pollutant interaction under the action of light promotes chemical processes (such as deethylation, demethylation, N-oxidation reactions, among others) to remove groups from the molecules of these contaminants. The other steps involve the cleavage of selective bonds (such as C–N and C–S), and addition reactions of −OH and =O [202,203,204,205].

In an overall analysis, the experimental validations discussed here, regarding the efficiency of the use of new metal oxide nanocomposites (MON) in water decontamination processes by photocatalysis, are essentially one of the first steps for the scalability of the solutions and for the adaptation of current wastewater treatment plants. A more integrated management of wastes is also under study (involving the various agents and related sectors): it is based on the possibility of expanding environmental services used in wastewater treatment plants and upgrading them to meet current decontamination demands [206]. Quite recently, literature reports have shown that the complexity of technological and economic decisions that must be considered for this type of expansion process can be better managed with the support of machine learning algorithms and circular bioeconomy techniques [206,207,208,209].

In general, machine learning methods can provide a quantitative assessment of the feasibility of adapting water treatment plants to new methodologies for decontamination of water polluted by organics, as well as the energy balance and the modernization needs of the sector. These actions aim to evaluate the economic returns generated by the new investments, combined with urban sustainability [206,207]. In addition, the search for new MON-based photocatalysts for wastewater treatment can contribute to a more progressive development of a circular bioeconomy, with innovative decontamination processes, to the emergence of intersectoral organizations, to greater interaction between research and legislation, to new methods of performance evaluation, and to a greater discussion of improvements aimed at society [206,208].

## 5. Conclusions

The fundamentals of metal oxides were presented and related to their physical and chemical characteristics. The electronic properties of those materials were described, allowing one to understand their application as photocatalysts in the treatment of organics-contaminated water.

The bibliometric study proved to be an important tool to highlight the present scenario and temporal evolution of the research topic in the last decade, as well as the main materials that have been used in synergy with metal oxides for the development of more efficient photocatalysts. The possibility of synthesizing nanocomposites between two or more metal oxides, or between metal oxides and conducting polymers or carbon materials, was highlighted as a viable, efficient, and low-cost alternative to improve the performance of water decontamination processes by heterogeneous photocatalysis.

The discussion of recent advances on the use of metal oxide nanocomposites demonstrated that most water decontamination processes involving these nanocomposites are carried out with visible light. This shows that charge transfer in the final semiconductor is facilitated by the presence of additional energy levels resulting from the interaction at the electronic level of these materials, which directly originates lower electron/hole recombination rates and facilitates the charge transport. These results are attributed to the improvement of the electronic coupling and to the fast capture of excited electrons in the metal oxide nanocomposites, when compared to the use of the pristine oxides. Their unique properties associated with a variety of applications in environmental remediation provide an exciting platform of research for the coming years.

## Figures and Tables

**Table 1:** Recent research results on the use of mixed MO-based MON photocatalysts for degradation of antibiotics and pesticides molecules in water. Results with less than a 10% loss of efficiency with reuse cycles were considered.

Mixed MO-Based MON	Light Stimulus/ Amount of Catalyst	Pollutant/Initial Concentration/ % Degradation/Number of Cycles Reuse	[Ref.]
grain bi-phase Fe_3_O_4_/Bi_2_WO_6_ supported on g-C3N4 nanosheets	Visible light/ 100 mg	Tetracycline antibiotic/20 mg L^−1^/ 98% in 105 min/6 cycles reuse	[93]
grain bi-phase Cu_2_O/Fe_3_O_4_ supported on Fe MOFs	Visible light/ 50 mg	Ciprofloxacin antibiotic/20 mg L^−1^/ 99.2% in 105 min/5 cycles reuse	[115]
heterogeneous nanoleaves of Cu/Ni/Fe oxides	Visible light/ 25 mg	Tetracycline antibiotic/10 mg L^¯1^ 100% in 4 min/12 cycles reuse	[124]
grain bi-phase TiO_2_/ZnO heteronanostructures	UV light/ 512 mg	Tetracycline antibiotic/20 mg L^−1^/ 82% after 165 min/6 cycles reuse	[125]
Fe_3_O_4_ nanoparticles on Bi_2_O_4_ nanorods	Visible light/ 10 mg	Ibuprofen antibiotic/500 μM/ 100%, after 240 h/4 cycles reuse	[126]
grain three-phase ZnO/Al_2_O_3_/TiO_2_ heteronanostructures	UV light/ 100 mg	Ibuprofen antibiotic/60 mg L^−1^/ 95%, after 210 min/no reuse	[127]
CuO/ZnFe_2_O_4_ nanoparticles on BiOBr nanoplates	Visible light/ 150 mg	Levofloxacin antibiotic/25 mg L^−1^ 91% in 90 min/5 cycles reuse	[128]
flower-like Bi_5_O_7_I/Bi/Bi_2_WO_6_ decorated NiFe_2_O_4_ nanoparticles	Visible light/ 75 mg	Levofloxacin antibiotic/28 mg L^−1^/ 97.5% in 90 min/5 cycles reuse	[129]
coral-like MgO/Co_3_O_4_ spherical nanostructures	Visible light/ 50 mg L^−1^	Levofloxacin antibiotic/10 mg L^−1^/ 96.9% in 20 min/6 cycles reuse	[130]
grain bi-phase CuO/CdO supported onto bentonite nanoleads	Sunlight/ 400 mg L^−1^	Levofloxacin antibiotic/10 mg L^−1^/ 96.1% in 30 min/3 cycles reuse	[131]
ZrO_2_ nanoparticles coated on MoO_3_ nanoplates	Visible light/ 250 mg L^−1^	Diclofenac sodium antibiotic/n.i./ 91% in 120 min/5 cycles reuse	[132]
Co_3_O_4_ nanoparticles dispersed on MoO_3_ surface	Sunlight/ 150 mg	Imidacloprid insecticide/15 mg L^−1^/ 98% in 150 min/no reuse	[133]
Fe-doped anatase/brookite TiO_2_ heteronanostructures	UV light/ 1 g L^−1^	Simazine herbicide/1.73 × 10^−5^ M 65% in 180 min/no reuse	[134]
CeO_2_ nanoparticles/WO_3_ nanoplates heteronanostructures	Visible light/ 30 mg	Nitenpyram insecticide/n.i./ 100% in 180 min/no reuse	[135]
FeO/CoO nanoparticles loaded onto the TiO_2_ surface	UV light/ 100 mg	2,4,6-Trichlorophenol herbicide/25 mg L^−1^/100% in 180 min/no reuse; 2,4-Dichlorophenoxyacetic acid herbicide/25 mg L^−1^/100% in 120 min/no reuse	[136]
grain bi-phase TiO_2_-MoO_3_ heteronanostructures	Visible light/ 50 mg	Carbaryl and Fenoxycarb insecticides/60 mg L^−1^/100% in 60 min/no reuse	[137]
Li_2_MnO_3_@ZrO_2_ core-shell Heterostructures	Visible light/ 1 g L^−1^	Atrazine herbicide/50 mg L^−1^/ 100% in 60 min/5 cycles reuse	[138]
NiO/ZnO heterostructures embedded in the chitosan pores	Sunlight/ 30 mg	Malathion insecticide/20 mg L^−1^/ 94% in 5 h/5 cycles reuse	[139]

n.i.—no information.

**Table 2:** Metal oxide/carbon materials-based MON photocatalysts for removing pollutants from water. Results with less than 10% loss of efficiency with reuse cycles were considered.

Metal Oxide/Carbon Material-Based MON	Light Light Stimulus/ Amount of Catalyst	Pollutant/Initial Concentration/ Pollutant/Initial Concentration/ % Degradation/Number of Cycles Reuse	[Ref.]
Cu_2_O/G Nanostructures	Visible light/ XXX mg	MB dye/900 mg L^−1^/94%, after 180 min/3 cycles reuse	[19]
TiO_2_/G (supported on clinoptilolite nanoplates)	Visible light (xenon lamp)	Nitenpyram insecticide/n.i./100%, after 80 min/no reuse	[169]
TiO_2_ on G surface	Visible light (tungsten lamp)/30 mg	RhB dye/30 mg L^−1^/84% in 6 h/ 2 cycles reuse	[173]
TiO_2_ on G surface	Visible light (xenon lamp)/10 mg	Xanthate (pollutant from the mineral industry)/20 mg L^−1^/97%, after 100 min/no reuse	[174]
TiO_2_/MoS_2_ on G surface	Visible light/n.i.	Tetracycline antibiotic/10 mg L^−1^/95%, after 60 min/no reuse	[175]
sandwich-structured N-doped (ZnO/G/ZnO) nanosheets	Visible light (xenon lamp)/300 mg	MOrange dye/10 mg L^−1^/80%, after 80 min/no reuse	[178]
V_2_O_5_ nanorods on G surface	Visible light (sunlight)/10 mg	MB dye/n.i/100% in 90 min/no reuse	[179]
WO_3_ nanorods on G nanosheets	visible light (xenon lamp)/20 mg	MB dye/10 mg L^−1^/83% in 70 min/no reuse	[180]
G nanocluster decorated Nb_2_O_5_ Nanofibers	Visible light (metal-halide lamp)/1 g	MOrange dye/20 mg L^−1^/95% in 5 h/ 3 cycles reuse	[181]
single-walled CNT on Mn_3_O_4_-TiO_2_ surface	Visible light (sunlight)/1 g	MOrange dye/20 mg L^−1^/98% in 150 min/no reuse	[182]
WO_3_.ZnO.NiO on CNT	Visible light (sunlight)/50 mg	MB dye/5 ppm/66.19% in 105 min/ 4 cycles reuse	[26]
multi-walled CNT decorated V-doped TiO_2_	Visible light (sunlight)/500 mg	MB dye/12.8 mg L^−1^/65%, after 60 min/no reuse	[167]
TiO_2_ nanoribbons/multi-walled CNT nanostructures	Visible light (sunlight)/20 mg	MB dye/10 mg L^−1^/97.3%, after 180 min/3 cycles reuse	[183]
TiO_2_/ZnO covered multi-walled CNT	UV; visible light/n.i.	RhB dye/5 mg L^−1^/100% in 40 min/no reuse	[184]
TiO_2_/CNTs/reduced graphene oxide (rGO) nanostructures	Visible light (xenon lamp)/10 mg	RhB dye/10 mg L^−1^/100%, after 60 min/no reuse	[185]
WO_3_ on multi-walled CNT surface	Visible light (xenon lamp)/50 mg	Naphthalene insecticide/10 ppm/65% in 240 min/no reuse	[186]
V_2_O_5_ on multi-walled CNT surface	UV light/10 mg	MB dye/100 ppm/96%, after 60 min/no reuse	[187]
CeO_2_@C_60_ core-shell nanostructures	Visible light (sunlight)/1 g	P-nitroaniline/10 ppm/100% in 75 min/7 cycles reuse	[188]
TiO_2_ and CeO_2_ nanofibers embedded in C_60_ nanowhiskers matrix	UV light/75 mg	Isopropyl alcohol (IPA)/200 ppm/ 90%, after 120 min/no reuse	[189]
Bi_2_TiO_4_F_2_-C_60_ hierarchical Spheres	Visible light (xenon lamp)/10 mg	RhB and Eosin Y/20 ppm/80% and 90%, respectively, after 60 min/ 3 cycles reuse	[190]

G—graphene; CNT—carbon nanotubes; C_60_—fullerene; MB—methylene blue; RhB—rhodamine B; MOrange—methyl orange; n.i.—no information.

## Data Availability

Not applicable.
